# Effects of new 17α-hydroxylase/C_17,20_-lyase inhibitors on LNCaP prostate cancer cell growth in vitro and in vivo

**DOI:** 10.1038/sj.bjc.6690739

**Published:** 1999-10

**Authors:** D N Grigoryev, B J Long, I P Nnane, V C O Njar, Y Liu, A M H Brodie

**Affiliations:** Department of Pharmacology and Experimental Therapeutics, University of Maryland, School of Medicine, Baltimore, 21201, USA

**Keywords:** 17α-hydroxylase/C_17,20_-lyase inhibitors, azolyl steroids, androgens synthesis, LNCaP cells, SCID mice

## Abstract

Our laboratory has been developing new inhibitors of a key regulatory enzyme of testicular and adrenal androgen synthesis 17α-hydroxylase/C_17,20_-lyase (P450c17), with the aim of improving prostate cancer treatment. We designed and evaluated two groups of azolyl steroids: Δ5-non-competitive inhibitors (Δ5NCIs), VN/63-1, VN/85-1, VN/87-1 and their corresponding Δ4 derivatives (Δ4NCIs), VN/107-1, VN/108-1 and VN/109-1. The human P450c17 gene was transfected into LNCaP human prostate cancer cells, and the resultant LNCaP-CYP17 cells were utilized to evaluate the inhibitory potency of the new azolyl steroids. VN/85-1 and VN/108-1 had the lowest IC_50_ values of 1.25 ± 0.44 nM and 2.96 ± 0.78 nM respectively, which are much lower than that of the known P450 inhibitor ketoconazole (80.7 ± 1.8 nM). To determine whether the compounds had direct actions on proliferation of wild-type LNCaP cells, cell growth studies were performed. All of the Δ5NCIs and VN/108-1 blocked the growth-stimulating effects of androgens. In steroid-free media, the Δ5NCIs decreased the proliferation of LNCaP cells by 35–40%, while all of the Δ4NCIs stimulated LNCaP cells growth 1.5- to 2-fold. In androgen receptor (AR) binding studies, carried out to determine the mechanism of this effect, all of the Δ4NCIs (5 μM) displaced 77–82% of synthetic androgen R1881 (5 nM) from the LNCaP AR. The anti-androgen flutamide and the Δ5NCIs displaced 53% and 32–51% of R1881 bound to AR respectively. These results suggested that the Δ5NCIs may also be acting as anti-androgens. We further evaluated our inhibitors in male severe combined immuno- deficient mice bearing LNCaP tumour xenografts. In this model VN/85-1 was as effective as finasteride at inhibiting tumor growth (26% and 28% inhibition, respectively) and the inhibitory effect of VN/87-1 was similar to that of castration (33% and 36% inhibition respectively). These results suggest that VN/85-1 and VN/87-1 may be potential candidates for treatment of prostate cancer. © 1999 Cancer Research Campaign


*British Journal of Cancer* (1999) **81**(4), 622–630
© 1999 Cancer Research Campaign
Article no. bjoc.1999.0739

### Effects of new 17α-hydroxylase/$C_{17,20}$-lyase inhibitors on LNCaP prostate cancer cell growth in vitro and in vivo

**DN Grigoryev, BJ Long, IP Nnane, VCO Njar, Y Liu and AMH Brodie**

Department of Pharmacology and Experimental Therapeutics, University of Maryland, School of Medicine, Baltimore, MD 21201, USA

**Summary** Our laboratory has been developing new inhibitors of a key regulatory enzyme of testicular and adrenal androgen synthesis 17α-hydroxylase/$C_{17,20}$-lyase (P450c17), with the aim of improving prostate cancer treatment. We designed and evaluated two groups of azolyl steroids: Δ5-non-competitive inhibitors (Δ5NCIs), VN/63-1, VN/85-1, VN/87-1 and their corresponding Δ4 derivatives (Δ4NCIs), VN/107-1, VN/108-1 and VN/109-1. The human P450c17 gene was transfected into LNCaP human prostate cancer cells, and the resultant LNCaP-CYP17 cells were utilized to evaluate the inhibitory potency of the new azolyl steroids. VN/85-1 and VN/108-1 had the lowest $IC_{50}$ values of 1.25 ± 0.44 nM and 2.96 ± 0.78 nM respectively, which are much lower than that of the known P450 inhibitor ketoconazole (80.7 ± 1.8 nM). To determine whether the compounds had direct actions on proliferation of wild-type LNCaP cells, cell growth studies were performed. All of the Δ5NCIs and VN/108-1 blocked the growth-stimulating effects of androgens. In steroid-free media, the Δ5NCIs decreased the proliferation of LNCaP cells by 35–40%, while all of the Δ4NCIs stimulated LNCaP cells growth 1.5- to 2-fold. In androgen receptor (AR) binding studies, carried out to determine the mechanism of this effect, all of the Δ4NCIs (5 μM) displaced 77–82% of synthetic androgen R1881 (5 nM) from the LNCaP AR. The anti-androgen flutamide and the Δ5NCIs displaced 53% and 32–51% of R1881 bound to AR respectively. These results suggested that the Δ5NCIs may also be acting as anti-androgens. We further evaluated our inhibitors in male severe combined immuno-deficient mice bearing LNCaP tumour xenografts. In this model VN/85-1 was as effective as finasteride at inhibiting tumor growth (26% and 28% inhibition, respectively) and the inhibitory effect of VN/87-1 was similar to that of castration (33% and 36% inhibition respectively). These results suggest that VN/85-1 and VN/87-1 may be potential candidates for treatment of prostate cancer. © 1999 Cancer Research Campaign

**Keywords:** 17α-hydroxylase/$C_{17,20}$-lyase inhibitors; azolyl steroids; androgens synthesis; LNCaP cells; SCID mice

Prostate cancer is the most common malignancy and the second leading cause of cancer-related deaths among men in the US and the UK (Shibata et al, 1998). Increased awareness of the disease and improved methods for early detection allow treatment to start when the tumour is still androgen-sensitive. Androgen ablation remains the most effective therapy for the treatment of prostate cancer. However, studies conducted by the National Cancer Institute (Crawford et al, 1992) and the European Organization for Research and Treatment of Cancer (Denis et al, 1994) showed that while the ablation of testicular androgens by chemical castration was an effective treatment option, inhibiting the action of adrenal androgens with anti-androgens significantly prolonged the survival of subset of prostate cancer patients. Therefore, complete instead of partial withdrawal of androgens could be an effective approach for the treatment of prostate cancer.

With this aim, our laboratory has designed enzyme inhibitors to block androgen synthesis from all sites (Ling et al, 1997; Njar et al, 1998). Steroidal 17α-hydroxylase/$C_{17,20}$-lyase (P450c17) (EC 1.14.99.0) was selected as the target enzyme for inhibition, as this is a key regulatory enzyme of androgen synthesis. P450c17 catalyses two sequential reactions in the steroid biosynthetic pathway and converts pregnenolone and progesterone to the C-19 androgens, dehydroepiandrosterone and androstenedione respectively. This enzyme has an identical amino acid sequence in testicular and adrenal tissues (Chung et al, 1987). Therefore, P450c17 inhibitors would be equally effective at both sites. In order to evaluate the effectiveness of these inhibitors we developed a multi-step system, which utilized the LNCaP human prostate cancer cell line. LNCaP cells remain the only androgen receptor (AR)-positive prostate cancer cell line, that can be readily grown in tissue culture (Horoszewicz et al, 1983). These cells respond to androgens with increased cell proliferation and elevated expression of prostate specific antigen (PSA) (Berns et al, 1993). LNCaP cells at early passages are not only androgen-responsive, but also androgen-dependent (Pousette et al, 1997). This is similar to the situation in most tumours of prostatic cancer patients, which are initially highly responsive and dependent on androgens. Although LNCaP cells have a mutated AR (Veldscholte et al, 1990), this cell line has been used extensively in research on the causes, treatment and prevention of prostate cancer (Weber et al, 1997). Therefore, we employed LNCaP cells as a model for evaluating the effectiveness of our new inhibitors of androgen synthesis.

In this study the compounds selected for evaluation were two groups of azolyl steroids. The first group has a double bond at the fifth carbon atom in the steroid molecule: the Δ5-non-competitive inhibitors (Δ5NCIs) of P450c17 are VN/63-1, VN/85-1 and VN/87-1. The second group are their corresponding Δ4 derivatives (Δ4NCIs), and are VN/107-1, VN/108-1 and VN/109-1, which have a double bond at the fourth carbon atom in the steroid molecule (Figure 1) (Njar et al, 1998).

Here we report on the application of LNCaP-CYP17-transfected cells for determining the inhibitory potencies of the new compounds against the P450c17 enzyme. As we have reported

*Received 8 January 1999*
*Revised 22 March 1999*
*Accepted 31 March 1999*

*Correspondence to:* AMH Brodie

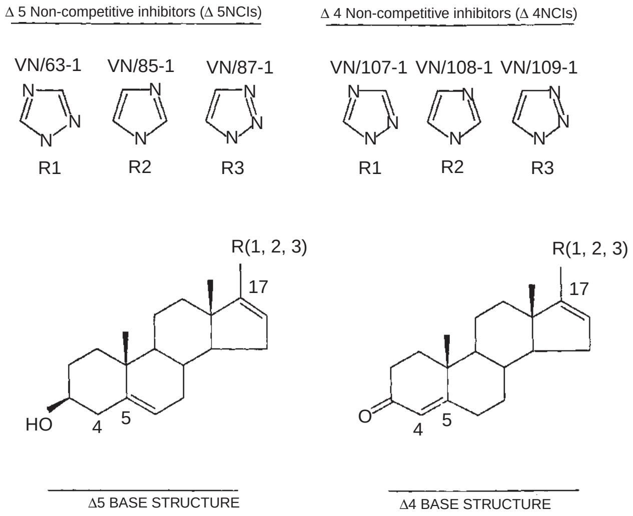

**Figure 1** Chemical structures of azolyl steroid inhibitors of 17α-hydroxylase/$C_{17,20}$-lyase. Compounds are divided in two groups: Δ5 non-competitive (left) and Δ4 non-competitive (right). All compounds in the same group have a common base structure and differ in the azolyl group at carbon position 17

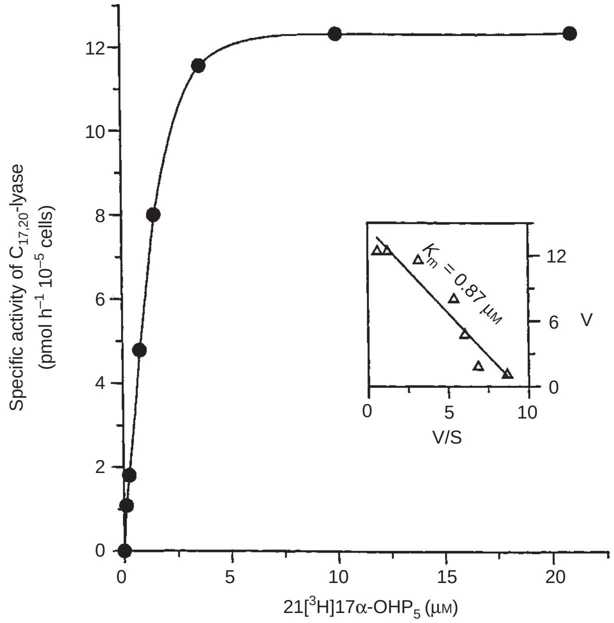

**Figure 2** Saturation curve of 17α-hydroxylase/$C_{17,20}$-lyase enzyme and Scatchard–Eadie plot (insert). LNCaP cells expressing the P450c17 enzyme were treated with different concentrations of [21$^3$H]17α-hydroxypregnenolone (0.5–20 μM). The specific activity was determined after incubation for 16 h at 37°C. Maximum activity (12.5 pmol $h^{-1}$ $10^{-5}$ cells) was reached at 7 μM concentration and $K_m$ was estimated by using RADLIG software as 0.87 μM (insert)

previously this cell line was the most suitable host among the tested mammalian cells for expression of functional P450c17 enzyme, and the $IC_{50}$ values of azolyl steroids obtained using this model were comparable to those obtained with human testicular microsomes (Grigoryev et al, 1999). We also utilized wild-type LNCaP prostatic carcinoma cells for evaluating the direct effects of new azolyl steroids on proliferation of these cells in vitro and in vivo.

### MATERIALS AND METHODS

### Materials

The human prostate cancer cell line LNCaP was obtained from the American Type Culture Collection (Rockville, MD, USA). RPMI-1640 medium, trypsin–EDTA (0.25%/0.02%), penicillin/streptomycin (P/S), geniticin (G418), and LipofectAMINE were from Gibco-BRL (Grand Island, NY, USA). Fetal bovine serum (FBS) was obtained from Summit Biotechnology Inc. (Fort Collins, CO, USA) and steroid-free FBS was prepared as described previously (Klus et al, 1996). Phenol red free IMEM and trypsin–versine were purchased from Biofluids Inc. (Rockville, MD, USA). Chloroform and acetonitrile were purchased from JT Baker, Inc (Phillipsburg, NJ, USA). Hydroxypropyl cellulose (HPC) was purchased from Sigma Chemical Co. (St Louis, MO, USA). The PSA enzyme-linked immunusorbent assay (ELISA) kit was from DSL Inc. (Webster, TX, USA). Matrigel was generously supplied by Dr Hynda Kleinman (NIH, Bethesda, MD, USA).

Inhibitors of androgen synthesis: VN/63-1, VN/85-1, VN/87-1, VN/107-1, VN/108-1 and VN/109-1 were synthesized in our laboratory as previously described (Njar et al, 1998). Ketoconazole was a gift from Dr Y Ling (Beijing Medical University, Beijing, China). Finasteride was kindly provided by Merck Research Laboratories (Rahway, NJ, USA). Flutamide was purchased from Sigma Chemical Co. [21-$^3$H]17α-Hydroxypregnenolone ([21-$^3$H]17αOHP$_5$: specific activity 13.61 mCi mmol$^{-1}$) was prepared in our laboratory as described previously (Njar et al, 1998). Metribolone [17α-methyl-$^3$H]-R1881 ([$^3$H]-R1881: specific activity 86 Ci mmol$^{-1}$) was purchased from Dupont NEN (Boston, MA, USA).

### Methods

#### *Transfection of LNCaP prostate cancer cells with pCDNA3Hmod17His*

LNCaP cells (passage number 26–30) were grown in RPMI-1640 medium containing 10% FBS and 1% P/S. LNCaP cells were transfected with the pCDNA3Hmod17His construct as we have previously described (Grigoryev et al, 1999) and grown in the same medium supplemented with 750 μg ml$^{-1}$ geniticin (G418). The surviving colonies were maintained in selective media and tested twice a month for $C_{17,20}$-lyase activity as described in the following section. After 3 months (16 passages) the transfectant cell line with the greatest $C_{17,20}$-lyase activity was selected (LNCaP-CYP17) and used for evaluation of $IC_{50}$ values of the prospective inhibitors.

#### *[$^3$H]-acetic acid releasing assay with LNCaP-CYP17*

The acetic acid releasing assay (AARA) was performed essentially as described previously (Grigoryev et al, 1999). Briefly, LNCaP-CYP17 cells were grown in 6-well tissue culture plates to 80% confluency. Cells were washed with Dulbecco's phosphate-buffered saline (DPBS), and incubated with steroid-free medium (1 ml) containing 0.5–20 μM of [21-$^3$H]17α-OHP$_5$. This substrate is converted to DHEA and [$^3$H]acetic acid is released during cleavage of the C-21 side chain. Cold 17α-hydroxypregnenolone (17α-OHP$_5$, 1 mM) was added to control wells for detection of non-specific substrate conversion. After a 16-h incubation at 37°C, medium was collected and the steroids were extracted with 2 ml of chloroform at 4°C. The aqueous phase, which contains the [$^3$H]acetic acid, was collected, and charcoal suspension was added to 2.5% final concentration. Following a 30-min incubation at 4°C, an aliquot of the supernatant was removed and radioactivity measured by liquid scintillation counting. $K_m$ and $V_{max}$ values were calculated using RADLIG 40 software (Biosoft, Ferguson, MO, USA). To determine the $IC_{50}$ values of the test compounds, LNCaP cells were incubated with medium (1 ml) containing 7 µM of [21-$^3$H]17α-$OHP_5$ (the saturating concentration; Figure 2) and different concentrations (1–100 nM) of the inhibitors. After a 16-h incubation, the medium was analysed as described above and $IC_{50}$ values were calculated.

### *Effects of the azolyl steroids and reference drugs on wild-type LNCaP cell growth*

LNCaP cell were transferred into steroid-free medium 3 days prior to the start of all experiments. Steroid-free medium consisted of phenol red-free IMEM supplemented with 5% steroid-free FBS and 1% P/S. Cells were then plated into 24-well culture plates (15 000 cells per well) in 1 ml of the same medium. After a 24-h attachment period, the vehicle (ethanol) or selected inhibitors at concentrations 0.5, 1.0, 2.5 and 5.0 µM were added to the triplicate wells. Medium and treatments were changed every 72 h. After 9 days of treatment, the cells were removed with trypsin–EDTA and counted in a Coulter counter (Coulter Electronics, Hialeah, FL, USA). The effects of the inhibitors on testosterone (0.1 nM) or DHT (0.03 nM) stimulated LNCaP cell growth were evaluated by the same method. For determining toxicity of compounds tested, LNCaP cells ($10^5$) were incubated in steroid-free media (1 ml) containing 0.5–20 µM of the test inhibitors. After 3 days, cells were collected and counted as described above. The toxicity of the compound I-49 was used as a positive control (Klus et al, 1996).

### *Androgen receptor binding assays*

Binding of [$^3$H]R1881 to the AR in the presence of the azolyl steroids and reference drugs was performed as described previously (Wong et al, 1995) with slight modifications. Briefly, 24-well plates were pre-treated with poly-L-lysine (0.05 mg $ml^{-1}$) for 30 min and dried. LNCaP cells were prepared as described above and plated at 2–3 × $10^5$ cells per well. After a 24-h incubation, culture medium was replaced with serum-free, phenol red-free IMEM containing 5 nM [$^3$H]R1881. To determine non-specific binding a 200-fold excess of cold R1881 was added to control wells. The test compounds (5 µM) were added to triplicate wells. Following a 2-h incubation at 37°C, the cells were washed twice with ice-cold DPBS and solubilized with 0.5% sodium dodecyl sulphate (SDS) and 20% glycerol in the same buffer. Extracts were removed and cell associated [$^3$H]R1881 was determined using a scintillation counter.

### *Evaluation of the test compounds in male SCID mice*

Male severe combined immunodeficient (SCID) mice were purchased from Charles River Breeding Laboratories (Boston, MA, USA). The animals were housed in a pathogen-free environment under controlled conditions of light and humidity and received food and water ad libitum. LNCaP tumours were grown subcutaneously (s.c.) in the mice essentially as described by Sato et al (1997). Briefly, wild-type LNCaP cells were grown in routine medium as described earlier. When 80% confluent, the cells were scraped into DPBS, counted, and suspended in Matrigel (3 × $10^7$ cells/ml). Male SCID mice were injected s.c. with 100 µl of the cell suspension at one site on each flank. LNCaP tumours were allowed to grow for 4–5 weeks following cell inoculation. The mice were then grouped (six mice per group) for castration or treatment with vehicle or the test inhibitors (50 mg $kg^{-1}$ $day^{-1}$). Tumours were measured weekly for the 4 weeks of treatment and tumour volumes were calculated using the formula $0.5236 \times r_1^2 \times r_2$ ($r_1 < r_2$). At the end of the treatment period, the animals were sacrificed by decapitation and blood collected. Serum was obtained after centrifugation and stored at –80°C. Tumours were excised, weighed and stored in liquid nitrogen.

**Table 1** Inhibition of human 17α-hydroxylase/$C_{17,20}$-lyase and 5α-reductase by novel azolyl steroids

| Compound | $IC_{50}$ (nM ± s.e.m.) | |
|---|---|---|
| | $C_{17,20}$-lyase | 5α-reductase |
| Ketoconazole | 80.7 ± 1.8 | NI |
| Finasteride | NI | 33 ± 2.0 |
| Flutamide | NI | NT |
| VN/63-1 | 47.1 ± 0.2 | NI |
| VN/85-1 | 1.25 ± 0.44 | NI |
| VN/87-1 | 7.97 ± 0.98 | NI |
| VN/107-1 | 12.4 ± 1.1 | 152 ± 10.0 |
| VN/108-1 | 2.96 ± 0.78 | 142 ± 5.0 |
| VN/109-1 | 5.39 ± 0.12 | 198 ± 33.0 |

NI, the $IC_{50}$ values are higher than 5000 nM; NT, not tested. The inhibitory effects of the listed compounds on activity of the human 17α-hydroxylase/$C_{17,20}$-lyase expressed in LNCaP-CYP17 cells were determined as described in Materials and Methods. Inhibition of 5α-reductase activity in human prostatic microsomes was reported previously (Njar et al, 1998). The inhibitory potency of the test compounds is expressed as $IC_{50}$. Values from three different experiments are expressed as mean ± s.e.m.

### *Measurement of serum PSA, testosterone and DHT levels*

Serum PSA concentrations were measured using a PSA ELISA kit (DSL Inc). Before assay, serum was thawed and diluted 1:10 in DPBS. PSA levels were determined in a 25 µl aliquot of diluted serum according to the manufacturers protocol. For measurement of serum testosterone and DHT levels, mouse serum was assayed according to the manufacturer's protocol with a $^{125}$I-testosterone RIA kit and a $^{125}$I-DHT RIA kit respectively (both kits were obtained from DSL Inc.). Radioactivity was counted using a Packard Cobra II gamma counter.

### RESULTS

### Inhibition of P450c17 activity in LNCaP-CYP17 cells by the azolyl steroids and reference drugs

The structures of compounds evaluated are shown in Figure 1. The $V_{max}$ of P450c17 in LNCaP-CYP17 cells was determined by AARA to be 11 pmol $10^{-5}$ cells $h^{-1}$ and $K_m$ was 0.87 µM (Figure 2). The effects of the inhibitors on P450c17 activity in this mammalian expression system are shown in Table 1. The $IC_{50}$ values of compounds tested were in the range 1–100 nM. The Δ5NCI VN/85-1 and its Δ4 derivative VN/108-1 were the most potent inhibitors of the enzyme activity, with $IC_{50}$ values of 1.25 nM and 2.96 nM respectively. This is consistent with our previously reported results obtained with human testicular microsomes and P450c17-transformed bacteria (Grigoryev et al, 1999). The $IC_{50}$

© 1999 Cancer Research Campaign

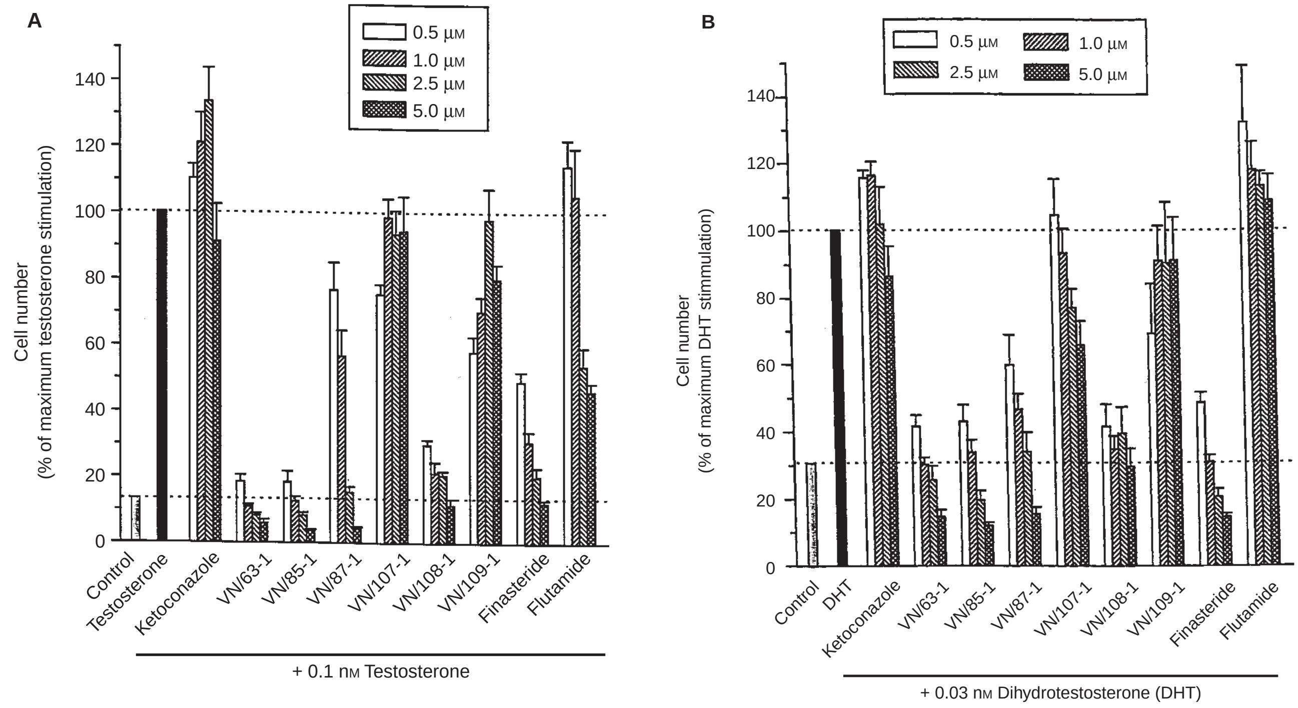

**Figure 3** Effects of the azolyl steroids and reference drugs on testosterone (**A**) and DHT (**B**) stimulated LNCaP cell proliferation. The ability of the compounds to inhibit the stimulatory effects of 0.1 nM testosterone or 0.03 nM DHT on LNCaP cell proliferation was tested as described in Materials and Methods. The columns represent the mean of the cell numbers in triplicate wells from three different experiments after incubation for 9 days. Cell number is expressed as a percentage of the mean number of the androgen stimulated cells. Values are expressed as mean ± s.e.m.

values for all of the new azolyl steroids were in the range 1.25–47.1 nM, and these values are much lower than that of the P450 inhibitor ketoconazole (80.7 nM). Table 1 also shows the previously reported $IC_{50}$ values of the compounds for human prostatic 5α-reductase (Nnane et al, 1998). All of the Δ4NCIs were effective against 5α-reductase but their $IC_{50}$ values were four- to sixfold higher than that of finasteride. However, the Δ5NCIs did not inhibit 5α-reductase.

### Effects of the azolyl steroids and reference drugs on LNCaP cell growth

The abilities of the test compounds to inhibit proliferation in wild-type LNCaP cells stimulated by 0.1 nM testosterone or 0.03 nM DHT was examined. We have previously reported that these concentrations of the androgens are most suitable for LNCaP growth stimulation studies (Klus et al, 1996). Testosterone stimulated LNCaP cell proliferation by 5.8-fold compared to the vehicle-treated control cells (Figure 3A). The growth stimulatory effect of testosterone was completely inhibited by 1.0 μM of VN/63-1 and VN/85-1, and by 2.5 μM of VN/87-1. VN/108-1 and finasteride were equally as effective at 5-μM concentration. VN/107-1 was ineffective against testosterone-stimulated cell growth and VN/109-1 had a moderate inhibitory effect. Ketoconazole was found to stimulate cell proliferation at low concentrations. However, both ketoconazole and VN/109-1 elicited biphasic growth responses. The anti-androgen flutamide inhibited testosterone stimulated cell growth by ~50% at concentrations of 2.5 and 5.0 μM (Figure 3A).

Cells were also treated with the inhibitors in the presence of 0.03 nM DHT. This concentration of androgen stimulated LNCaP cell proliferation by 3.2-fold compared to vehicle-treated cells (Figure 3B). DHT stimulation was completely blocked by 1.0 μM of VN/63-1, VN/85-1 and finasteride; 2.5 μM of VN/87-1; and 5.0 μM of VN/108-1. VN/107-1 and VN/109-1 were again found to be the least effective inhibitors of androgen-induced cell proliferation. Ketoconazole and flutamide stimulated cell growth by 10–20% compared to DHT-treated cells (Figure 3B).

To investigate whether the compounds may be acting as anti-androgens, LNCaP cells were treated with the inhibitors alone in steroid-free media for 9 days (Figure 4). All of the Δ5NCIs inhibited cell growth in a concentration-dependent manner by 40–60%. In contrast, all of the Δ4NCIs stimulated cell growth by 1.5 to 2-fold. Finasteride at concentrations of 1.0–5.0 μM inhibited cell growth by 20–30%. High concentrations of ketoconazole (2.5 and 5.0 μM) stimulated cell growth by 30–40%. Flutamide stimulated cell growth approximately sixfold in a biphasic manner (Figure 4). None of the novel compounds were found to be toxic to the LNCaP cells at the concentrations tested.

### LNCaP androgen receptor binding assays

The ability of the test compounds to prevent binding of the synthetic androgen R1881 to LNCaP AR was examined. LNCaP cells were incubated in steroid-free media containing 5 nM [$^3$H]R1881 and 5 μM of the test compounds and reference drugs. All of the Δ4NCIs were more potent than the anti-androgen flutamide at displacing [$^3$H]R1881 from the LNCaP AR. Flutamide displaced 50% of [$^3$H]R1881, while the Δ4NCIs displaced 70–80% (Figure 5). The Δ5NCIs VN/63-1, VN/85-1 and

© 1999 Cancer Research Campaign 

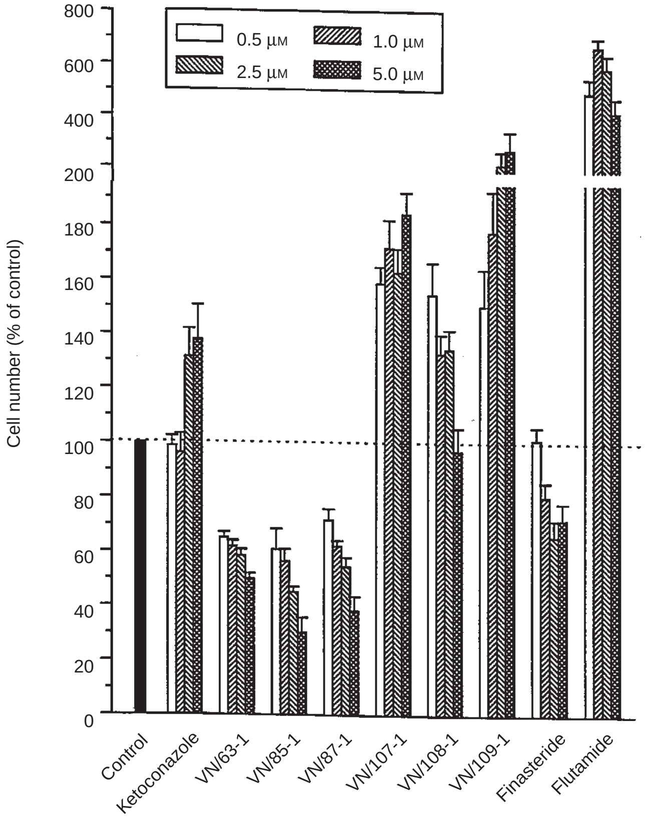

**Figure 4** Effects of the azolyl steroids and reference drugs on LNCaP cell proliferation in steroid-free medium. LNCaP cells were exposed to the test compounds in steroid-free media. After 9 days, cells were collected and counted as described in Materials and Methods. The columns represent the mean of the cell numbers in triplicate wells from three different experiments. Cell number is expressed as a percentage of the mean number in the control wells. Values are expressed as mean ± s.e.m.

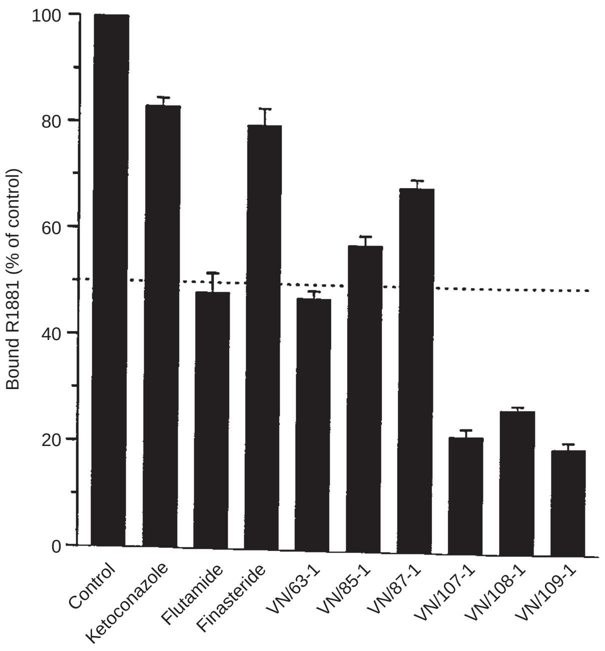

**Figure 5** Competition of the azolyl steroids with the synthetic androgen R1881 for the LNCaP androgen receptor. LNCaP cells were incubated in steroid-free media containing 5 nM of R1881 with or without 5 µM of the inhibitors. After incubation for 2 h, medium was removed and cells were washed with DPBS. Cells were collected and bound R1881 was determined as described in Materials and Methods. The columns show the percent of R1881 (pmol per cell) bound to the cells and are expressed as a percentage of total bound R1881. Values from three different experiments are expressed as mean ± s.e.m.

VN/87-1 displaced 50%, 40% and 30% of [$^3$H]R1881 respectively. Ketoconazole and finasteride displaced 17% and 20% of [3H]R1881 respectively (Figure 5).

### Effects of the inhibitors in LNCaP tumour growth in male SCID mice

Male SCID mice bearing LNCaP tumour xenografts were treated with the azolyl steroids (50 mg kg$^{-1}$ day$^{-1}$) for 4 weeks and the tumour volumes were measured weekly. In each of the experiments ketoconazole, finasteride and castration were used as reference groups. In the first experiment the effects of VN/85-1, VN/87-1 and VN/108-1 on LNCaP tumour growth were examined (Figure 6A). VN/108-1 inhibited LNCaP tumour growth by only 9%. VN/85-1 inhibited tumour growth by 25% and in this model was as effective as finasteride, which inhibited tumour growth by 27%. VN/87-1 inhibited tumour growth by 33%, which was similar to the result obtained with the castrated mice (36% growth inhibition). These reductions in tumour volumes correlated with final tumour weights (Figure 6B). Tumour weights in the castrated group were the lowest (68% of control). Tumour weights in the VN/87-1 group were reduced by 31% compared to the vehicle-treated mice and were reduced by 22% and 27% in the animals treated with finasteride and VN/85-1 respectively. Serum PSA levels were significantly lower in the mice treated with castration, finasteride, VN/87-1 ($P < 0.02$) VN/85-1 ($P < 0.04$) and surprisingly VN/108-1 ($P < 0.02$) (Figure 6B). Serum androgen levels in castrated mice and in the mice treated with VN/85-1, VN/87-1 and VN/108-1 were significantly reduced (Table 2). Serum DHT levels were also significantly lower in the mice treated with finasteride ($P < 0.01$).

**Table 2** Serum levels of testosterone and DHT in male SCID mice following 28 days of treatment with the azolyl steroids and reference drugs

| Treatment group | Testosterone (ng ml$^{-1}$) | DHT (pg ml$^{-1}$) |
|---|---|---|
| Control | 1.043 ± 0.120 | 243.8 ± 21.5 |
| Castrate | 0.286 ± 0.045[a] | 162.0 ± 12.3[c] |
| Ketoconazole | 1.526 ± 0.456 | 224.3 ± 31.9 |
| Finasteride | 1.389 ± 0.107 | 137.0 ± 7.7[b] |
| VN/85-1 | 0.141 ± 0.028[a] | 143.7 ± 13.2[b] |
| VN/87-1 | 0.343 ± 0.042[b] | 128.6 ± 4.9[a] |
| VN/108-1 | 0.081 ± 0.015[b] | 122.1 ± 6.4[a] |

[a]$P < 0.001$ versus control; [b]$P < 0.01$ versus control; [c]P < 0.05 versus control.

In the second experiment the effects of VN/63-1, VN/107-1 and VN/109-1 on LNCaP tumour growth were examined (Figure 7A). LNCaP tumour growth was found to be stimulated 110% by VN/109-1, when compared to control mice. Neither VN/63-1, VN/107 nor ketoconazole had any appreciable effects on the growth of the tumours following 28 days of treatment. The only treatments that reduced tumour volumes were finasteride (29% growth inhibition) and castration (33% growth inhibition). These results were also

 © 1999 Cancer Research Campaign

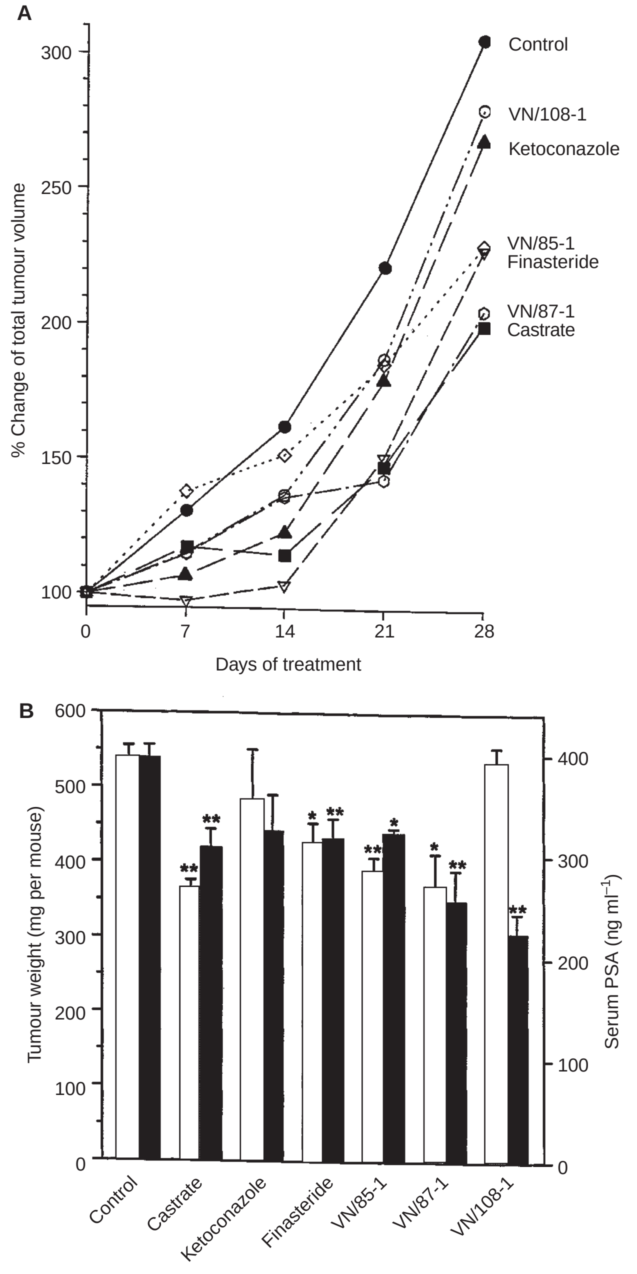

**Figure 6** Effects of the VN/85-1, VN/87-1, VN/108-1 and reference drugs on the growth of LNCaP tumour in male SCID mice and serum PSA levels. LNCaP cells were inoculated s.c. into SCID mice and allowed to proliferate as tumour xenografts to 300 $mm^3$. Groups of 5–6 mice were injected daily with the test compounds (50 mg $kg^{-1}$ $day^{-1}$). (**A**) Tumours were measured weekly and percentage change in volumes were calculated. (**B**) After 28 days of treatment, mice were sacrificed, tumours were weighed and serum PSA levels measured. The open bars represent the tumour weights (mg per mouse) and closed bars represent mean serum concentrations of prostate specific antigen (ng $ml^{-1}$). Values are expressed as mean ± s.e.m. (*$P < 0.04$, **$P < 0.02$)

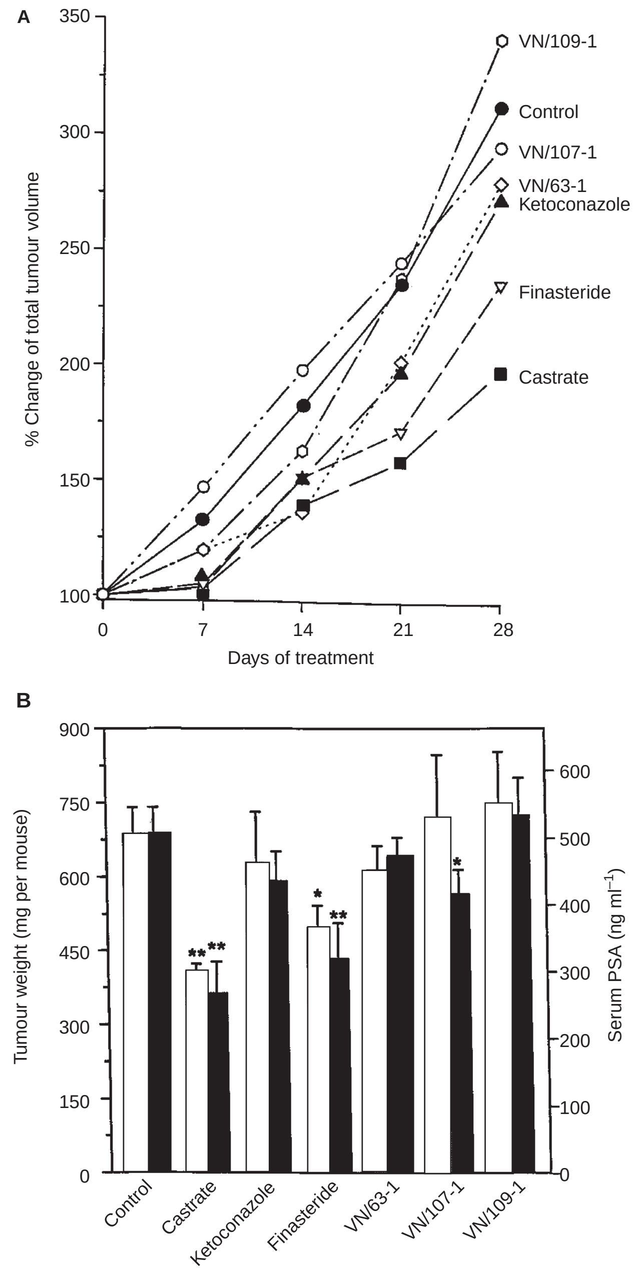

**Figure 7** Effects of the VN/63-1, VN/107-1, VN/109-1 and reference drugs on the growth of LNCaP tumor in male SCID mice and serum PSA levels. Procedures were the same as indicated in Figure 6

reflected in final tumour weights (Figure 7B). Tumour weights in the castrated group were reduced by 41%, and in the finasteride-treated group by 28%. In agreement with the result obtained in the first experiment, serum PSA levels were significantly lower in the mice treated with castration and finasteride ($P < 0.04$). Serum testosterone levels were significantly reduced in the mice treated with castration ($P < 0.001$) and VN/109-1 ($P < 0.05$); and significantly higher in the mice treated with finasteride, which was expected because inhibition of 5α-reductase often results in increased circulating levels of testosterone (Gormley et al, 1992). Serum DHT levels were significantly lower ($P < 0.05$) in the group treated with VN/107-1 (Table 2).

### DISCUSSION

There have been several reports on the importance of maximal androgen ablation for the treatment of prostate cancer (Crawford et al, 1989, 1992; Visakorpy et al, 1995). These studies reported that

**Table 3** Serum levels of testosterone and DHT in male SCID mice following 28 days of treatment with the azolyl steroids and reference drugs

| Treatment group | Testosterone (ng ml$^{-1}$) | DHT (pg ml$^{-1}$) |
|---|---|---|
| Control | 0.205 ± 0.015 | 239.2 ± 20.5 |
| Castrate | 0.054 ± 0.019[a] | 209.8 ± 13.4 |
| Ketoconazole | 0.298 ± 0.116 | 218.0 ± 43.4 |
| Finasteride | 0.638 ± 0.453[b] | 223.4 ± 19.6 |
| VN/63-1 | 0.146 ± 0.035 | 189.7 ± 17.9 |
| VN/107-1 | 0.153 ± 0.016 | 180.2 ± 8.7[b] |
| VN/109-1 | 0.564 ± 0.86[b] | 228.4 ± 42.9 |

[a]$P < 0.001$ versus control; [b]$P < 0.05$ versus control.

while ablation of testicular androgens by chemical castration is an effective treatment option, castration combined with anti-androgens to inhibit the action of adrenal androgens significantly prolonged the survival of prostate cancer patients. Moreover, patients who relapse on anti-androgen therapy may experience a second response upon anti-androgen withdrawal (Small et al, 1997). As an alternative approach to inhibiting the production of androgens from all sites, our laboratory has been developing inhibitors of P450c17, which is a key regulatory enzyme of the androgen biosynthetic pathway (Ling et al, 1997; Njar et al, 1998). P450c17 catalyses two sequential reactions in the steroid biosynthetic pathway. The 17α-hydroxylase reaction converts pregnenolone and progesterone to 17α-hydroxypregnenolone (17α-$OHP_5$) and 17α-hydroxyprogesterone (17α-$OHP_4$) respectively. Then, the $C_{17,20}$-lyase reaction converts 17α-$OHP_5$ and 17α-$OHP_4$ to the immediate androgen precursors dehydroepiandrosterone and androstenedione respectively. The known cytochrome P450 inhibitor ketoconazole has activity against P450c17 and has been used for the treatment of prostate cancer, both as a substitute for androgen ablation therapy (Trachtenberg et al, 1984) and as an adjuvant to castration (Williams et al, 1986). However, this drug has low P450c17 specificity, it inhibits cortisol production and is associated with high hepatotoxicity (Jubilerer et al, 1989). Nonetheless, Small et al (1997) have reported that ketoconazole administration is beneficial to patients with advanced prostate carcinoma who have relapsed from anti-androgen (flutamide) therapy. Using LNCaP prostate cancer cells transfected with the human P450c17 gene (CYP17), we determined that some of our new azolyl steroids were 50- to 60 fold stronger than ketoconazole at inhibiting P450c17 and that VN/85-1 and its Δ4 counterpart VN/108-1 were the most potent inhibitors (Table 1). Therefore, we hypothesized that these compounds would be effective for the treatment of prostate cancer.

In the present study we examined the abilities of several of our novel azolyl steroids to inhibit the growth of LNCaP prostate cancer cells in vitro and in vivo, and compared their effects to the known prostate cancer drugs finasteride, flutamide and ketoconazole. The compounds tested were the Δ5NCIs, VN/63-1, VN/85-1 and VN/87-1, and their Δ4 counterparts, VN/107-1, VN/108-1 and VN/109-1 (Njar et al, 1998). All of the Δ5NCIs, VN/108-1 and finasteride showed concentration-dependent inhibitory effects on testosterone stimulated LNCaP cell proliferation in culture, and VN/63-1 and VN/85-1 were the most potent inhibitors of testosterone induced cell growth (Figure 3A). This inhibition was unexpected, because these compounds were designed as inhibitors of the P450c17 enzyme, which is not expressed in LNCaP cells. The antiproliferative effects of the Δ5NCIs could not be due to 5α-reductase inhibition since these compounds have no effect on 5α-reductase activity in prostatic microsomes (Table 1) and displayed the same growth inhibitory effects on DHT-stimulated as on testosterone-stimulated LNCaP cell growth (Figure 3B). In addition, these effects were not due to a direct cytotoxic effect of the compounds on LNCaP cells (data not shown). These results suggest that, in addition to being potent inhibitors of P450c17, the Δ5NCIs may have other as yet undefined activities. VN/107-1, unlike its Δ5 counterpart (VN/63-1), had no antiproliferative activity. VN/109-1 retained approximately the same activity as its Δ5 analogue (VN/87-1) at concentrations of 0.5 and 1.0 μM, but had no inhibitory activity at higher concentrations. The anti-androgen flutamide, which has an androgenic effect on LNCaP cells (Wilding et al, 1989) unexpectedly inhibited testosterone-stimulated but not DHT-stimulated cell proliferation at high concentrations. This effect could be due to an androgen-like effect of the drug. It has been shown previously that LNCaP cells react to androgen stimulation with a biphasic concentration-dependent response, and that high concentrations of androgens can inhibit cell growth (De Launoit et al, 1991). The addition of flutamide may have resulted in an increase in total amount of AR agonists in testosterone (0.1 nM) supplied media to higher levels than in DHT (0.03 nM) supplied media. However, in clinical practice such a high concentration of flutamide cannot be achieved, and prolonged exposure to the drug has resulted in the proliferation of tumour cells that contain a mutant AR similar to LNCaP cells (Sartor et al, 1994).

We have previously reported that finasteride and some of our novel compounds may also be acting as anti-androgens against the LNCaP AR (Klus et al, 1996), and that this may be contributing to their antiproliferative activities. Moreover, our previous study also reported that finasteride is only an antagonist of the LNCaP AR although not of wild-type AR. We evaluated our new azolyl steroids for possible anti-androgenic effects by treating LNCaP cells in steroid-free medium with the inhibitors alone. At 5-μM concentration finasteride inhibited LNCaP cell growth by 25%. However, the Δ5NCIs were the most potent inhibitors of cell proliferation (Figure 4). The order of potency of the compounds was: VN/85-1 > VN/87-1 > VN/63-1. Cell growth was inhibited by 70%, 62% and 51% respectively. Similarly, all of the Δ5NCIs (5 μM) displaced the synthetic androgen R1881 from LNCaP AR with a higher potency than finasteride (Figure 5). The order of potency was VN/63-1 > VN/85-1 > VN/87-1 > finasteride. Flutamide was again found to stimulate LNCaP cell growth in steroid-free media by eightfold and the cells responded to the drug in a biphasic manner. All of the Δ4NCIs, and high concentrations of ketoconazole, also stimulated LNCaP cells proliferation (Figure 4). Moreover, the ability of the Δ4NCIs to displace R1881 from cellular AR was twofold higher than that of flutamide. The order of potency was VN/109-1 > VN/107-1 > VN/108-1 > flutamide. Interestingly, VN/108-1, which is a potent inhibitor of androgen-induced LNCaP cell proliferation, stimulated cell growth in absence of androgens, testosterone and DHT. The results presented here indicate that VN/108-1 is likely to stimulate LNCaP cell growth through binding to cellular AR. This finding characterizes VN/108-1 as a partial agonist of LNCaP AR.

In order to determine the effects of our new inhibitors in vivo, we utilized LNCaP prostate cancer cells grown as tumour xenografts in male SCID mice (Sato et al, 1997). The effectiveness of the inhibitors was evaluated by measuring changes in tumour volumes during a 4-week treatment period. Mice were treated with the compounds at 50 mg kg$^{-1}$ day$^{-1}$, and reference treatments were castration, ketoconazole and finasteride. VN/109-1 was ineffective

 © 1999 Cancer Research Campaign in this model, and moreover it stimulated LNCaP tumour growth (Figure 7). Although VN/107-1 and VN/108-1 reduced the circulating levels of androgens (Tables 2 and 3), these compounds had no significant effects on tumour growth or tumour weights (Figures 6 and 7). This implies that androgenic properties of these compounds outweigh their inhibitory effects on androgen synthesis. VN/63-1 reduced serum androgen levels, but this reduction did not gain statistical significance (Table 3) and its growth inhibitory potency was similar to that of ketoconazole (Figure 7). VN/85-1 was as effective as finasteride and VN/87-1 was as effective as castration in inhibiting tumour growth, and both compounds significantly reduced serum androgen levels (Table 2). Tumour weights were consistent with the tumour volume data. Tumours from castrated groups had the lowest weights in both experiments and were similar to the weights of the tumours in the mice treated with VN/87-1 and VN/85-1. The anti-tumour effects are consistent with the activity of these two compounds in other assays. However, these inhibitors of androgen synthesis were not more effective than castration at reducing serum androgen levels in the mice (Table 2). The reason castration was the most effective treatment in this study is unknown, but the results suggest that factors other than androgens may modulate LNCaP tumour growth in vivo.

PSA is widely used as a marker of prostatic cancer cell proliferation and, in this model, serum PSA levels were generally correlated with the weights of the tumours. Surprisingly, serum from the animals treated with VN/108-1 had the lowest levels of serum PSA (Figure 6B) and androgens (Table 2), despite the fact that this compound was ineffective in inhibiting tumour growth. As noted above, VN/108-1 appears to function as a partial agonist/antagonist of LNCaP AR. Thus, despite stimulating cell growth, it appears to down-regulate levels of PSA. Nevertheless, the results obtained with the reference treatments and novel compounds indicate that VN/85-1 and VN/87-1 have the most potent anti-tumour activity.

In this report we have described the use of an LNCaP cell system for evaluating six new inhibitors of P450c17. The screening protocol presented here demonstrates the useful application of the LNCaP prostate cancer cell line for evaluating new P450c17 inhibitors in vitro and in vivo. This system allows the evaluation of new compounds for activity against the P450c17 enzyme, their ability to interact with the AR and gives indirect evidence of activity against 5α-reductase. We have shown that VN/85-1 and VN/87-1, the most potent P450c17 inhibitors which are also anti-androgens, have potent anti-tumour effects in male SCID mice bearing LNCaP xenografts. However, further studies using other doses of the inhibitors and modes of administration are necessary to determine fully the usefulness of the compounds.

### ACKNOWLEDGEMENTS

We thank Dr. M. Waterman for the generous gift of pWCHmod17 construct containing human P450c17 gene. This work was supported by the NIH grant CA-27440.

### REFERENCES

Berns EMJJ, de Boer W and Mulder E (1993) Androgen-dependent growth regulation of and release of specific proteins by the androgen receptor containing human prostate tumor cell line LNCaP. *Prostate* **23**: 213–223

Chung B, Picado-Leonard J, Hahju M, Bienkowski M, Hall PF, Shively JE and Miller WL (1987) *Proc Natl Acad Sci USA* **84**: 407–411

Crawford ED, Eisenberg M, McLeod DG, Spaulding JT, Benson R, Dorr FA, Blumstein BA, Davis MA and Goodman PJ (1989) A controlled trial of leuprolide with and without flutamide in prostatic carcinoma. *N Engl J Med* **321**: 419–424

Crawford ED, Eisenberg M and McLeod DG (1992) Treatment of newly diagnosed stage D2 prostate cancer with leuprolide and flutamide or leuprolide alone, phase III, prognostic significance of minimal disease. *J Urol* **147**: 417A

De Launoit Y, Veilleux R, Dufour M, Simard J and Labrie F (1991) Characteristics of the biphasic action of androgens and of the potent antiproliferative effects of new pure antiestrogen EM-139 on cell cycle kinetic parameters in LNCaP human prostatic cancer cells. *Cancer Res* **51**: 5165–5170

Denis L (1994) Role of maximal androgen blockage in advanced prostate cancer. *Prostate* **5** (Suppl): 17–22

Gormley GJ (1991) Role of 5α-reductase inhibitors in the treatment of advanced prostatic carcinoma. *Urol Clin North Am* **18**: 93–97

Gormley GJ, Stoner E, Bruskewitz RC, Imperato-McGinley J, Walsh PC, McConnell JD, Andriole GL, Geller J, Bracken BR, Tenover JS, Vaughan ED, Pappas ED, Taylor A, Binkowitz B and Ng J (1992) The effect of finasteride in men with benign prostatic hyperplasia. *N Engl J Med* 1992 **327**: 1185–1191.

Grigoryev DN, Kato K, Njar VCO, Long BJ, Ling Y-z, Wang X, Mohler J and Brodie AMH (1999) Cytochrome P450c17 expressing *E.coli* as a first-step screening system for 17α-hydroxylase-$C_{17,20}$-lyase inhibitors. *Analyt Biochem* **267**: 319–330

Horoszewicz JS, Lying SS, Kawinski E, Karr JP, Rosental H, Chu TM, Mirand EA and Murphy GP (1983) LNCaP model of human prostatic carcinoma. *Cancer Res* **43**: 1809–1818

Jubilerer SJ and Hogan T (1989) High dose ketoconazole for the treatment of hormone-refractory metastatic prostate cancer: 16 cases and review of the literature. *J Urol* **142**: 89–91

Klus GT, Nakamura J, Li J-s, Ling Y-z, Son C, Kemppainen JA, Wilson EM and Brodie AMH (1996). Growth inhibition of human prostate cells in vitro by novel inhibitors of androgen synthesis. *Cancer Res* **56**: 4956–4964

Ling Y, Li J, Liu Y, Kato K, Klus GT, Brodie AMH (1997) 17-Imidazolyl, pyrazolyl, and isoxazolyl androstene derivatives. Novel steroidal inhibitors of human cytochrome C17,20-lyase ($P450_{17\alpha}$). *J Med Chem* **40**: 3297–3304

Nakajin S, Hall PF and Onoda M (1981) Testicular microsomal P-450 for $C_{21}$ steroid side chain cleavage: spectral and binding studies. *J Biol Chem* **256**: 6134–6139

Njar VCO, Kato K, Nnane IP, Grigoryev DN, Long BJ and Brodie AMH (1998) Novel 17-azolyl steroids, potent inhibitors of human cytochrome 17α-hydroxylase-$C_{17,20}$-lyase ($P450_{17\alpha}$): potential agents for the treatment of prostate cancer. *J Med Chem* **41**: 902–912

Pousette A, Carlstrom K, Henriksson P, Grande M and Stege R (1997) Use of a hormone-sensitive (LNCaP) and a hormone-resistant (LNCaP-r) cell line in prostate cancer research. *Prostate* **31**: 198–203

Sartor, O, Cooper M, Weinberger M, Headlee D, Thibault A, Tomkins A, Steinberg S, Figg WD, Linehan WM and Myers CE (1994) Surprising activity of flutamide withdrawal, when combined with aminoglutethimide in treatment of 'hormone-refractory' prostate cancer. *J Natl Cancer Inst* **86**: 222–227

Sato N, Gleave ME, Bruchovsky N, Rennie PS, Beraldi E and Sullivan LD (1997) A metastatic and androgen-sensitive human prostate cancer model using intraprostatic inoculation of LNCaP cells in SCID mice. *Cancer Res* **57**: 1584–1589

Schuurmans AL, Bolt J, Veldscholte J and Mulder E (1991) Regulation of growth of LNCaP human prostate tumor cells by growth factors and steroid hormones. *J Steroid Biochem Mol Biol* **40**: 193–197

Shibata A, Ma J and Whittemore AS (1998) Prostate cancer incidence and mortality in the United States and the United Kingdom. *J Natl Cancer Inst* **90**: 1230–1231

Small EJ, Baron A and Bok R (1997) Simultaneous antiandrogen withdrawal and treatment with ketoconazole and hydrocortisone in patients with advanced prostate carcinoma. *Cancer* **80**: 1755–1759

Trachtenberg J and Pont A (1984) Ketoconazole therapy for advanced prostatic cancer. *Lancet* **2**: 433–435

Veldscholte J, Ris-Stalpers C, Kuiper GGJM, Jenster G, Berrevoets C, Claassen E, Van Rooij HCJ, Trapman J, Brinkmann AO and Mulder E (1990) A mutation in the ligand-binding domain of the androgen receptor of human LNCaP cells affects steroidal binding characteristics and response to androgens. *Biochem Biophys Res Comman* **173**: 534–540

Veldscholte J, Berrevoets C, Brinkmann AO, Grootegoed JA and Mulder E (1992) Androgens and the mutated androgen receptor of LNCaP cells: differential effects on binding affinity, heat-shock protein interaction and transcription activation. *Biochem* **31**: 2393–2399

Visakorpy T, Hyytinen E, Koivisto P, Tanner M, Keinanen R, Palmberg C, Palotie A, Tammela T, Isola J and Kallioniemi O-P (1995) In vivo amplification of the androgen receptor gene and progression of human prostate cancer. *Nat Genet* **9**: 401–406

© 1999 Cancer Research Campaign 

Webber MM, Bello D and Quader S (1997) Immortalized and tumorigenic adult human prostatic epithelial cell lines: characteristics and applications Part 2. Tumorigenic cell lines. *Prostate* **30**: 58–64
Wilding G, Chen M and Gellman EP (1989) Aberrant response in vitro of hormone responsive prostate cancer cells to antiandrogens. *Prostate* **14**: 103–115
Williams G, Kerle DJ, Doble A, Dunlop H, Smith C, Allen J, Yeo T and Bloom SR (1986) Objective responses to ketoconazole therapy in patients with relapsed progressive prostatic cancer. *Br J Urol* **58**: 45–51
Wong C, Kelce WR, Sar M and Wilson EM (1995) Androgen receptor antagonist versus agonist activities of the fungicide vinclozin relative to hydroxyflutamide. *J Biol Chem* **270**: 19998–20003

 *© 1999 Cancer Research Campaign*

## References

[PDF_00954] Chung B. C., Picado-Leonard J., Haniu M., Bienkowski M., Hall P. F., Shively J. E., Miller W. L. (1987). Cytochrome P450c17 (steroid 17 alpha-hydroxylase/17,20 lyase): cloning of human adrenal and testis cDNAs indicates the same gene is expressed in both tissues.. Proc Natl Acad Sci U S A.

[PDF_00956] Crawford E. D., Eisenberger M. A., McLeod D. G., Spaulding J. T., Benson R., Dorr F. A., Blumenstein B. A., Davis M. A., Goodman P. J. (1989). A controlled trial of leuprolide with and without flutamide in prostatic carcinoma.. N Engl J Med.

[PDF_00967] Denis L. (1994). Role of maximal androgen blockade in advanced prostate cancer.. Prostate Suppl.

[PDF_00969] Gormley G. J. (1991). Role of 5 alpha-reductase inhibitors in the treatment of advanced prostatic carcinoma.. Urol Clin North Am.

[PDF_00971] Gormley G. J., Stoner E., Bruskewitz R. C., Imperato-McGinley J., Walsh P. C., McConnell J. D., Andriole G. L., Geller J., Bracken B. R., Tenover J. S. (1992). The effect of finasteride in men with benign prostatic hyperplasia. The Finasteride Study Group.. N Engl J Med.

[PDF_00976] Grigoryev D. N., Kato K., Njar V. C., Long B. J., Ling Y. Z., Wang X., Mohler J., Brodie A. M. (1999). Cytochrome P450c17-expressing Escherichia coli as a first-step screening system for 17alpha-hydroxylase-C17,20-lyase inhibitors.. Anal Biochem.

[PDF_00980] Horoszewicz J. S., Leong S. S., Kawinski E., Karr J. P., Rosenthal H., Chu T. M., Mirand E. A., Murphy G. P. (1983). LNCaP model of human prostatic carcinoma.. Cancer Res.

[PDF_00983] Jubelirer S. J., Hogan T. (1989). High dose ketoconazole for the treatment of hormone refractory metastatic prostate carcinoma: 16 cases and review of the literature.. J Urol.

[PDF_00986] Klus G. T., Nakamura J., Li J. S., Ling Y. Z., Son C., Kemppainen J. A., Wilson E. M., Brodie A. M. (1996). Growth inhibition of human prostate cells in vitro by novel inhibitors of androgen synthesis.. Cancer Res.

[PDF_00951] Langeler E. G., van Uffelen C. J., Blankenstein M. A., van Steenbrugge G. J., Mulder E. (1993). Effect of culture conditions on androgen sensitivity of the human prostatic cancer cell line LNCaP.. Prostate.

[PDF_00989] Ling Y. Z., Li J. S., Liu Y., Kato K., Klus G. T., Brodie A. (1997). 17-Imidazolyl, pyrazolyl, and isoxazolyl androstene derivatives. Novel steroidal inhibitors of human cytochrome C17,20-lyase (P450(17 alpha).. J Med Chem.

[PDF_00993] Nakajin S., Hall P. F., Onoda M. (1981). Testicular microsomal cytochrome P-450 for C21 steroid side chain cleavage. Spectral and binding studies.. J Biol Chem.

[PDF_00996] Njar V. C., Kato K., Nnane I. P., Grigoryev D. N., Long B. J., Brodie A. M. (1998). Novel 17-azolyl steroids, potent inhibitors of human cytochrome 17 alpha-hydroxylase-C17,20-lyase (P450(17) alpha): potential agents for the treatment of prostate cancer.. J Med Chem.

[PDF_01002] Pousette A., Carlström K., Henriksson P., Grande M., Stege R. (1997). Use of a hormone-sensitive (LNCaP) and a hormone-resistant (LNCaP-r) cell line in prostate cancer research.. Prostate.

[PDF_01005] Sartor O., Cooper M., Weinberger M., Headlee D., Thibault A., Tompkins A., Steinberg S., Figg W. D., Linehan W. M., Myers C. E. (1994). Surprising activity of flutamide withdrawal, when combined with aminoglutethimide, in treatment of "hormone-refractory" prostate cancer.. J Natl Cancer Inst.

[PDF_01009] Sato N., Gleave M. E., Bruchovsky N., Rennie P. S., Beraldi E., Sullivan L. D. (1997). A metastatic and androgen-sensitive human prostate cancer model using intraprostatic inoculation of LNCaP cells in SCID mice.. Cancer Res.

[PDF_01013] Schuurmans A. L., Bolt J., Veldscholte J., Mulder E. (1991). Regulation of growth of LNCaP human prostate tumor cells by growth factors and steroid hormones.. J Steroid Biochem Mol Biol.

[PDF_01016] Shibata A., Ma J., Whittemore A. S. (1998). Prostate cancer incidence and mortality in the United States and the United Kingdom.. J Natl Cancer Inst.

[PDF_01019] Small E. J., Baron A., Bok R. (1997). Simultaneous antiandrogen withdrawal and treatment with ketoconazole and hydrocortisone in patients with advanced prostate carcinoma.. Cancer.

[PDF_01022] Trachtenberg J., Pont A. (1984). Ketoconazole therapy for advanced prostate cancer.. Lancet.

[PDF_01029] Veldscholte J., Berrevoets C. A., Brinkmann A. O., Grootegoed J. A., Mulder E. (1992). Anti-androgens and the mutated androgen receptor of LNCaP cells: differential effects on binding affinity, heat-shock protein interaction, and transcription activation.. Biochemistry.

[PDF_01024] Veldscholte J., Ris-Stalpers C., Kuiper G. G., Jenster G., Berrevoets C., Claassen E., van Rooij H. C., Trapman J., Brinkmann A. O., Mulder E. (1990). A mutation in the ligand binding domain of the androgen receptor of human LNCaP cells affects steroid binding characteristics and response to anti-androgens.. Biochem Biophys Res Commun.

[PDF_01033] Visakorpi T., Hyytinen E., Koivisto P., Tanner M., Keinänen R., Palmberg C., Palotie A., Tammela T., Isola J., Kallioniemi O. P. (1995). In vivo amplification of the androgen receptor gene and progression of human prostate cancer.. Nat Genet.

[PDF_01042] Webber M. M., Bello D., Quader S. (1997). Immortalized and tumorigenic adult human prostatic epithelial cell lines: characteristics and applications Part 2. Tumorigenic cell lines.. Prostate.

[PDF_01045] Wilding G., Chen M., Gelmann E. P. (1989). Aberrant response in vitro of hormone-responsive prostate cancer cells to antiandrogens.. Prostate.

[PDF_01048] Williams G., Kerle D. J., Ware H., Doble A., Dunlop H., Smith C., Allen J., Yeo T., Bloom S. R. (1986). Objective responses to ketoconazole therapy in patients with relapsed progressive prostatic cancer.. Br J Urol.

[PDF_01051] Wong C., Kelce W. R., Sar M., Wilson E. M. (1995). Androgen receptor antagonist versus agonist activities of the fungicide vinclozolin relative to hydroxyflutamide.. J Biol Chem.

[PDF_00963] de Launoit Y., Veilleux R., Dufour M., Simard J., Labrie F. (1991). Characteristics of the biphasic action of androgens and of the potent antiproliferative effects of the new pure antiestrogen EM-139 on cell cycle kinetic parameters in LNCaP human prostatic cancer cells.. Cancer Res.

